# Lipoic Acid Metabolism as a Potential Chemotherapeutic Target Against *Plasmodium falciparum* and *Staphylococcus aureus*

**DOI:** 10.3389/fchem.2021.742175

**Published:** 2021-11-04

**Authors:** Sun Liu Rei Yan, Felipe Wakasuqui, Xiaochen Du, Matthew R. Groves, Carsten Wrenger

**Affiliations:** ^1^ Unit for Drug Discovery, Department of Parasitology, Institute of Biomedical Sciences–ICB, University of São Paulo, São Paulo, Brazil; ^2^ Structural Biology in Drug Design, Department of Drug Design, Groningen Research Institute of Pharmacy, University of Groningen, Groningen, Netherlands

**Keywords:** Lipoic acid, malaria, protein lipoylation, *Plasmodium*, *S*. *aureus*, Lipoic acid (LA), lipoylation

## Abstract

Lipoic acid (LA) is an organic compound that plays a key role in cellular metabolism. It participates in a posttranslational modification (PTM) named lipoylation, an event that is highly conserved and that occurs in multimeric metabolic enzymes of very distinct microorganisms such as *Plasmodium sp.* and *Staphylococcus aureus*, including pyruvate dehydrogenase (PDH) and *α*-ketoglutarate dehydrogenase (KDH). In this mini review, we revisit the recent literature regarding LA metabolism in *Plasmodium sp.* and *Staphylococcus aureus*, by covering the lipoate ligase proteins in both microorganisms, the role of lipoate ligase proteins and insights for possible inhibitors of lipoate ligases.

## Introduction

**FIGURE 1:**
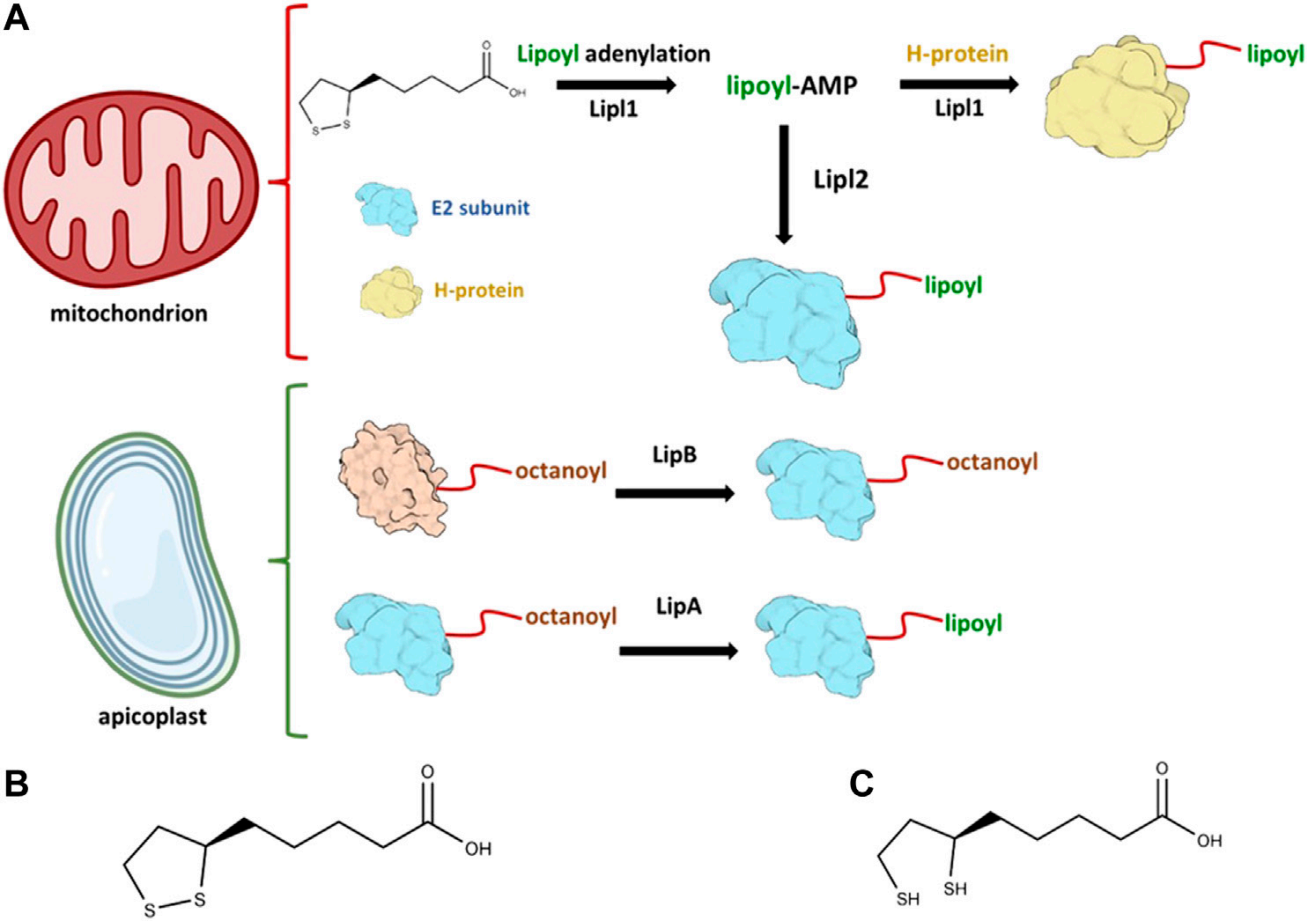
Lipoylation in *Plasmodium falciparum* occurs in both mitochondrion and apicoplast. In **(A)** the enzymatic reactions that takes place in each of these compartments are illustrated. The generation of lipoyl-AMP is needed to activate Lipl2 and lipoylate the E2 subunit of both BCDH and KDH. The oxidized lipoate is also attached to the H-protein of the parasite. Oxidized lipoic acid is shown in **(B)** and reduced lipoic acid is shown in **(C)**, Illustrations were created with BioRender.com (License #2364–1,511, Toronto, ON, Canada).

LA {6,8-dithiooctanoic acid or 5-[(3*R*)-dithiolan-3-yl]pentanoic acid} is an organosulfur compound (Figure 1B and Figure 1C) that has long been reported for antioxidant effects and potential therapeutic benefits in treating a variety of diseases, such as neurodegenerative diseases, diabetes, and cardiovascular conditions (Marangon et al., 1999; Amom et al., 2008; McNeilly et al., 2011; Tromba et al., 2019; Li et al., 2020; Molz et al., 2021). In addition to its potential therapeutic effects and current use as a potential antioxidant in dietary supplementation, LA is an essential cofactor for many enzymatic reactions in key biochemical pathways. To date, LA is known to act as a cofactor in five different enzyme complexes: the glycine cleavage system (GCS), pyruvate dehydrogenase (PDH), *α*-ketoglutarate dehydrogenase (KGDH), branched-chain *α*-keto acid dehydrogenase (BCDH), and acetoin dehydrogenase (AoDH) (Oppenheim et al., 2014). The *α*-ketodehydrogenase-complexes contain three protein subunits, named E1, E2, and E3. LA in the free form of lipoate is attached to the E2 lysine residues or to the H protein of the GCS. Lipoate metabolism is present across different human pathogens, including *Plasmodium sp.*, the causative agent of malaria, a tropical disease that was responsible for about 229 million cases worldwide in 2019 only, with an estimated 409,000 deaths in the same year (World Health Organization, 2020). Although more knowledge on lipoylation has been gained (Cao et al., 2018a; Laczkovich et al., 2018; Zhang et al., 2020; Tang et al., 2021), it remains an attractive topic to better understand the metabolic consequences of dysregulated lipoylation and how LA metabolism enzymes could be explored as a potential drug target in different diseases. From a chemical perspective, the disulfide bond in oxidized/reduced form provides a strong redox couple that is important for reactive oxygen species (ROS) scavenging and for the redox-dependent reactions that regulate multienzyme complexes. Examples of scavenged ROS include peroxynitrite (ONOO−), hypochlorous acid (HClO), peroxyl radical (ROO·), and hydroxyl radicals (·OH). However, evidence so far indicates that hydrogen peroxide (H_2_O_2_) is not directly scavenged by LA (Xiao et al., 2012). LA can also act as a chelator of Cu^2+^, Pb^2+^ and Zn^2+^ *in vitro*. Therefore, LA could be potentially a treatment for diseases where these metals may play an important role in their progression (Xiao et al., 2012; Hane and Leonenko, 2014; Smirnova et al., 2018).

### Lipoylation in *Plasmodium falciparum*

Antimalarial drug resistance still is a major health concern worldwide and poses a real threat for the control of malaria (WHO, 2020). Two main species are responsible for the majority of malaria cases worldwide: *P. vivax* represents 75% of malaria cases in the Americas, while 99.7% of estimated malaria cases in Africa were caused by *P. falciparum* (World Health Organization, 2021). Lipoylation of *Plasmodium* proteins is an event that occurs in two different compartments of the parasite: mitochondrion and the apicoplast, a unique *Apicomplexan* plastid organelle that evolved from endosymbiotic events. The parasite relies both on lipoate biosynthesis and lipoate-scavenged pathways: lipoate biosynthesis takes place in the apicoplast, while scavenged lipoate from host is metabolized in the mitochondrion.

Two important *P. falciparum* lipoate ligases, Lipl1 and Lipl2, are known to play key roles in protein lipoylation. Lipl1 is responsible for the lipoylation of GcvH protein by employing the oxidized form of lipoate and Lipl2 is responsible for the lipoylation of the E2 subunit from BCDH and KDH. However, Lipl2 depends on the formation of dihydrolipoyl-AMP in reducing conditions to transfer the lipoyl moiety to the N-lysine residue, thereby impacting the activity of Lipl1 in a reducing environment. In oxidative decarboxylation reactions of *α*-ketoacid complexes, lipoate derived from LA plays a key role as a cofactor. The *α*-ketoacids complexes are formed by three different subunits, named E1, E2, and E3. In the case of the glycine cleavage complex (GCV), the H-protein (GcvH) serves as the lipoyl domain for lipoate. All three proteins–GcvH, E2 subunit of both BCDH and KDH (E2-BCDH and E2-KDH, respectively)–are lipoylated through lipoate covalent ligation to the lysine residue at the N-terminus of lipoate domain and they are known to be localized in the parasite mitochondrion (Afanador et al., 2014). *In vitro* lipoylation assays show that Lipl1 is required for the lipoylation of GcvH, E2-BCDH and E2-KDH (Afanador et al., 2014). Evidence so far indicates that the role of Lipl2 in *P. falciparum* is to act as a lipoyl-AMP:*N* ^ε^-lysine lipoyltransferase. The intermediate lipoyl-AMP generated by Lipl1 is employed by Lipl2 to lipoylate both E2-BCDH and E2-KDH. Therefore, the catalytic activity of Lipl2 also depends on the activity of Lipl1, since only Lipl1 can generate lipoyl-AMP conjugate (Afanador et al., 2014). Recently, Leung and colleagues et al (Leung et al., 2021) discussed the role of GcvH beyond GCV system, such as its possible role in the lipoylation of *α*-ketoacids dehydrogenase proteins. Although recent work has been performed to better elucidate the LA metabolism in eukaryotes (Cao et al., 2018a; Vacchina et al., 2018; Biddau et al., 2021; Pietikäinen et al., 2021), there are still unanswered questions, such as why there are two protein ligases instead of only one protein ligase in *Plasmodium* and the role of lipoylated proteins in the parasite. Lipoate scavenging for use in the mitochondrion remains an open research topic to be elucidated. Scientific evidence so far indicates that lipoate scavenged from the host is important for *P. falciparum* erythrocytic stage parasites (Günther et al., 2009a). Murine malaria models and human malaria model show that lipoylation may play an essential role in *Plasmodium* (Wang et al., 2017). In particular, an experiment in which LA analogues were utilized (Deschermeier et al., 2012) showed decreased mitochondrial lipoylation and inhibition of parasite growth. A general scheme of how proteins are lipoylated in *P. falciparum* is briefly described in Figure 1A. Lipl1 is known to be a mitochondrial protein while Lipl2 is both mitochondrial and found in the apicoplast, a unique plastid organelle found in *Apicomplexan* parasites. This organelle is important in *Plasmodium sp.* due to the presence of important parasite metabolic pathways, such as the synthesis of isopentenyl diphosphate (IPP) (Wiley et al., 2015), a precursor of isoprenoids, the type II fatty acid synthesis (FAS-II) (van Schaijk et al., 2014), and the lipoate biosynthesis that is mediated by two different enzymes: octanoyl-ACP:protein *N-*octanoyltransferase (LipB) and lipoyl synthase (LipA) (Wrenger and Müller, 2004). LipB and LipA orchestrate the biosynthesis of LA in the apicoplast: LipB is responsible for the transfer of the octanoyl-moiety to the E2 subunit whereas LipA acts as a catalyst for the insertion of two sulfurs at positions C6 and C8 of the octanoyl-moiety that is bound to the E2 subunit of the PDH in the apicoplast.

Recently, Biddau and collaborators (Biddau et al., 2021) provided more evidence on the putative role of LA in redox regulation. Using a *N*-octanoyltransferase (LipB) *P. falciparum* 3D7 knockout strain, the authors identified upregulation of antioxidant-related cytosolic proteins that could be related to plastid-cytosol signaling. Additionally, experiments in *Anopheles* mosquitoes that are the vector for malaria transmission indicated that LipB knockout parasites could not produce salivary gland sporozoites, possibly indicating the need of LA synthesis in the apicoplast for the full development of *P. falciparum* in *Anopheles*.

**FIGURE 2:**
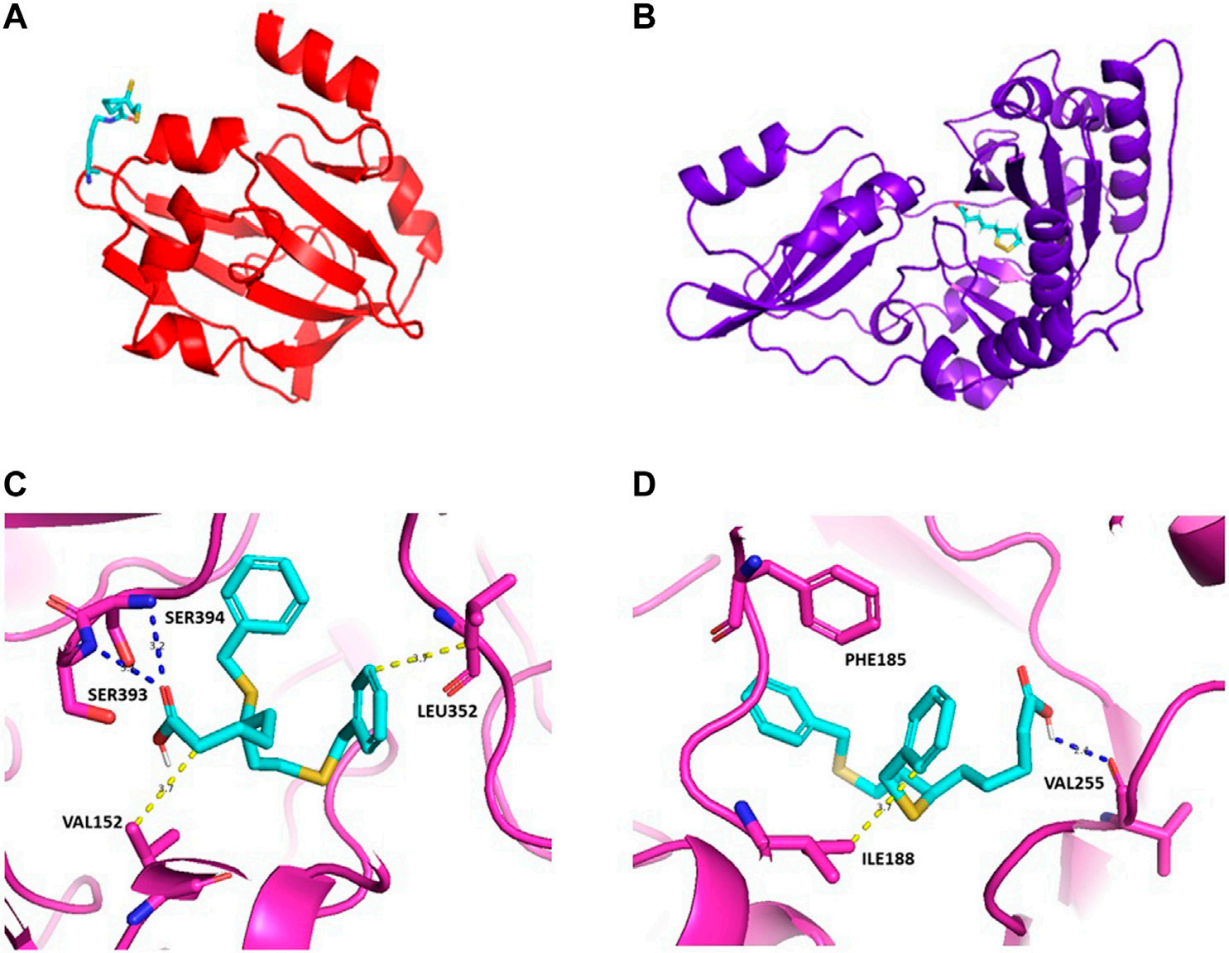
Homology models for *S. aureus* GcvH protein **(A)** and Lipl1 protein **(B)**. Lipoate moiety shown in stick representation with *PyMOL*. PDB access numbers: 3AB9 and 5T8U. Predicted three-dimensional structure for LipA of *P falciparum* with devimistat. The three dimensional structure of LipA was obtained from AlphaFold (PDB access number: Q8IDQ0), the search for a predicted active site was performed using the package FtMap (Kozakov et al., 2015). Based upon the two predicted binding sites docking was performed using Autodock/sMina. The lowest energy poses are shown in **(C)** and **(D)** for each predicted binding site. Potential hydrophobic interactions are shown by dotted yellow lines with the indicated residues, blue dotted lines represent hydrogen bonds; pi stacking: PHE185. In **(C)**, the calculated binding energy is of −7.312 kcal/mol and, in **(D)**, the calculated binding energy is of −7.075 kcal/mol.

In terms of drug discovery, the lack of the structural information available for LipA in *Plasmodium sp.* may also require *in silico* predictions, as the one available from Alphafold (Jumper et al., 2021). We used the FTMap web servers (Kozakov et al., 2015) for predicting protein binding hot spots through computational approaches, which shows that there are two potential hot spots, cluster 1 and cluster 2 [Figure 2C and 2(d)]. Devimistat, an LA analogue drug candidate, was chosen for investigation as a potential inhibitor compound. Molecular docking using AutoDock/Smina (Koes et al., 2013) was used to predict the binding-conformation of devimistat to LipA. The result shows devimistat bound to lipoic acid protein at the catalytic site of the homologous *α*-ketoglutarate dioxygenase of *E. coli* (RCSB: 1GY9, Figure 2C and 2(d). For cluster 1 (Figure 2C), there are two hydrogen bonds between serine 394, serine 393 and a compound carboxy groups hydrophobic interaction between leucine 352 and the compound 8-phenyl group, valine 152 and the compound 2-carbon atom. For cluster 2 (Figure 2D), there is a hydrogen bond between valine 255 and a compound carboxy functional group; hydrophobic interaction between isoleucine 188 and the compound 6-phenyl group; pi stacking between phenylalanine 185 and the compound 6,8-diphenyl groups.

### Fatty Acid Synthesis (FAS-II) and Iron-Sulfur (Fe-S) Clusters in *Plasmodium sp.*

Lipids are required for *Plasmodium* growth and replication. The FAS-II pathway is present in the malaria parasite apicoplast, specifically in sporozoite and liver stages. Vaughan and colleagues et al. Vaughan et al. (2009) demonstrated the importance of FAS-II for the parasite when migrating from liver to the blood asexual stage, both in *P. yoelli* and *P. falciparum*. Later it was also shown (van Schaijk et al., 2014) that FAS-II is required for midgut oocyst sporozoite production during the *Anopheles* mosquito stage of *P. falciparum* life cycle, but dispensable in rodent malaria models of *P. yoelli* and *P. berghei*. Thus, much research has been conducted to explore inhibitory approaches in the late-liver parasite development. While initial FAS-II inhibitors eliminated blood-stage malaria parasites, their mode of action were shown to be off-target (McFadden and Yeh, 2017). Most of these drugs act in the blood stage of the parasite cycle, yet FAS-II is not essential in that stage. Therefore, tackling the parasite at liver stage is likely to be a more promising avenue for drug development. In terms of lipoylation of *P. falciparum* proteins, the FAS-II pathway in the apicoplast has the fundamental role of providing the octanoyl-acyl carrier protein (octanoyl-ACP) as a precursor for the generation of *de novo* LA (Wrenger and Müller, 2004; Shears et al., 2015).

In *Plasmodium*, the importance of Fe-S clusters for intraerythrocytic stage growth is well established. Fe-S clusters are found in different forms, such as 4Fe-4S. These clusters can act as cofactors and bind proteins *via* cysteine residues. Two proteins of the *P. falciparum* sulfur mobilization pathway (SUF) were characterized (Charan et al., 2014): *Pf*SufS and *Pf*SufE. The first has cysteine desulfurase activity while the latter enhances the activity of *Pf*SufS. Both proteins mediate sulfur mobilization, which is the first step in the apicoplast SUF pathway, and both are localized in the apicoplast. Since SUF is not present in humans, the enzymes of this pathway are attractive targets for parasite inhibition and eventually the validation of this pathway for druggability purposes. More recently, functional experiments performed with *P. vivax* in clinical isolates show that the SUF pathway is conserved as in laboratory strains (Pala et al., 2019). As briefly described in this mini review, the lipoate synthase (LipA) is an example of an enzyme that depends on 4Fe-4S clusters. It is localized in the apicoplast, where it uses the iron sulfur clusters to add sulfur atoms onto carbons C6 and C8 of the octanoic acid, thereby completing the lipoate synthesis on the PDH E2 subunit (Thomsen-Zieger et al., 2003; Wrenger and Müller, 2004; Günther et al., 2009b; Storm and Müller, 2012; Shears et al., 2015).

### LA Metabolism in *Staphylococcus aureus*

*Staphylococcus aureus* is a gram-positive bacterium able to colonize human skin and mucous membranes, living as a commensal in healthy individuals. However, it is also capable of invasion, causing several clinical manifestations. It is a leading cause of endocarditis, bacteremia, osteomyelitis and skin and soft tissue infections (Turner et al., 2019). The greatest concern with *S. aureus* is its developed multi drug resistance and persistent high mortality (van Hal et al., 2012).

Like in *P. falciparum*, *S. aureus* has both *de novo* biosynthetic pathway and salvage pathway for the generation of LA. With the exception of *B. anthracis*, no other pathogenic *Firmicutes* has such a diversity of enzymes involved in the acquisition of this cofactor (Spalding and Prigge, 2010). *S. aureus* has two lipoate-protein ligases, named LplA1 and LplA2, for salvaging LA and octanoic acid from the environment (Zorzoli et al., 2016; Cao et al., 2018b). The attachment of free LA in *S. aureus* occurs *via* LplAs in a two-step reaction: first there is the formation of the intermediate lipoyl-AMP in the presence of Mg^2+^ and ATP, followed by the binding of the lipoyl group to the apoprotein (Fujiwara et al., 2005).

LplA1 binds LA mainly to GcvH, while LplA2 binds LA to the E2 subunits of *α*-ketoacid dehydrogenases, as well as to the operon-linked GcvH-like protein, GcvH-L (Laczkovich et al., 2018). GcvH and GcvH-L thereby provide storage of lipoylated proteins. *In vivo* studies using mouse infection models of LplA1 or LplA2 knockouts demonstrated that the presence of either enzyme is enough to promote kidney infection (Zorzoli et al., 2016). LplA2 is encoded in an operon together with an ADP-ribosyltransferase, macrodomain protein, luciferase-like monooxygenase and the protein GcvH-L, which suggests that LplA2 may participate in LA-dependent maintenance of redox homeostasis (Rack et al., 2015). Illustration of homology models for *S. aureus* GcvH and LplA1 proteins with lipoate moiety is shown in Figures 2A, B

Biosynthesis of LA begins when the fatty acid intermediate octanoyl is transferred from an acyl carrier protein to the *ε*-amino group of a lysine in the lipoyl domain of the GcvH by the enzyme LipM. (Douglas et al., 2006; Zorzoli et al., 2016). The octanoyl moiety is sulfurized into lipoyl by the enzyme LipA. LipA is a member of the radical *S*-adenosyl-l-methionine (SAM) enzymes (Christensen and Cronan, 2010), which use a [4Fe-4S] cluster as an electron donor to reductively cleave SAM, generating a deoxyadenosyl radical and methionine. Two 5-deoxyadenosyl break C-H bond in position 6 and 8 of the octanoyl moiety, creating carbon radicals that allow sulphur insertions, with the auxiliary iron-sulphur cluster of LipA acting as a sulphur donor (Douglas et al., 2006). Finally, *S. aureus* possess the enzyme LipL, an amido transferase responsible for the transfer of octanoyl or lipoyl groups between GcvH, GcvH-like protein (GcvH-L) and *α*-keto dehydrogenases, as well as inter E2-subunits. This flexibility gives *S. aureus* robust resources to supplement its requirements for LA (Teoh et al., 2019).

LA biosynthesis and its salvage pathway plays a major role in facilitating the pathogenesis of microorganisms, promoting pathogen survival within an infected host (Spalding and Prigge, 2010). *S. aureus* has specific requirements during infection, where *de novo* biosynthesis of LA is necessary to infect the heart and salvage is required for infection of the kidney (Zorzoli et al., 2016). LipL was shown to be necessary within the host, but not necessary at skin sites, where *S. aureus* can overcome its need for branched-chain fatty acids by scavenging unsaturated fatty acids from host skin. (Teoh et al., 2021). In addition, lipoyl-E2-PDH secreted in the extracellular environment blunts activation of macrophage toll-like 1/2 receptors (Grayczyk et al., 2017) and reduces the generation of ROS and RNS by macrophage NADPH oxidase and iNOS (Grayczyk and Alonzo, 2019), which reinforces the importance of lipoylation in the context of infection.

### LA Metabolism in Humans and Other Organisms

As mentioned above, lipoylation is a PTM event that occurs in different organisms. Good drug targets must have minimal effects on the host or at least mitigation of off-target effects. The catalysis of LA assembly in human was recently elucidated by Cao and colleagueset al. (Cao et al., 2018a), reporting the relevant LIPT1 and LIPT2 enzyme activities: LIPT1 catalyzes the attachment of the lipoyl moiety to the lipoyl domain of the protein, acting as a lipoyl amidotransferase, while LIPT2 acts as an octanoyltransferase. Both proteins are located in the mitochondria. In humans, the insertion of two sulfur atoms to generate the lipoyl moiety is catalyzed by the mitochondrial protein lipoyl synthase (LIAS) and utilizing [4Fe-4S] as a co-factor.

Lipoylation events in humans remains an open research topic, since the enzymes involved in the pathway may be potential drug targets against cancer, as recently demonstrated for CPI-613^®^ (devimistat) (Zachar et al., 2011; Gao et al., 2020), which is currently designated as an orphan drug by FDA for the treatment of metastatic pancreatic cancer. Lipoylation event is also present in other protozoans, for example *Trypanosoma cruzi* (Vacchina et al., 2018), where growth of parasite in medium liver infusion tryptose (LIT) with 10 fold lower glucose concentration (0.4 g/L) increased the lipoylated state of PDH E2 subunits. The LA analogue 8-bromo-octanoic acid showed inhibition of parasite growth in different protozoans, including *T. cruzi*, *P. falciparum*, and *Toxoplasma gondii* (Crawford et al., 2006; Allary et al., 2007).
